# Cardiac q‐space trajectory imaging by motion‐compensated tensor‐valued diffusion encoding in human heart in vivo

**DOI:** 10.1002/mrm.29637

**Published:** 2023-03-20

**Authors:** Irvin Teh, David Shelley, Jordan H. Boyle, Fenglei Zhou, Ana‐Maria Poenar, Noor Sharrack, Richard J. Foster, Nadira Y. Yuldasheva, Geoff J. M. Parker, Erica Dall'Armellina, Sven Plein, Jürgen E. Schneider, Filip Szczepankiewicz

**Affiliations:** ^1^ Leeds Institute of Cardiovascular and Metabolic Medicine University of Leeds Leeds UK; ^2^ Leeds Teaching Hospitals Trust Leeds UK; ^3^ Faculty of Industrial Design Engineering Delft University of Technology Delft Netherlands; ^4^ Center for Medical Image Computing, Department of Medical Physics & Biomedical Engineering and Department of Neuroinflammation University College London London UK; ^5^ Astrea Bioseparation Comberton UK; ^6^ Bioxydyn Limited Manchester UK; ^7^ Medical Radiation Physics, Clinical Sciences Lund Lund University Lund Sweden

**Keywords:** cardiac microstructure, diffusion tensor imaging, motion‐compensated diffusion encoding, q‐space trajectory imaging, tensor‐valued diffusion encoding, tissue characterization

## Abstract

**Purpose:**

Tensor‐valued diffusion encoding can probe more specific features of tissue microstructure than what is available by conventional diffusion weighting. In this work, we investigate the technical feasibility of tensor‐valued diffusion encoding at high *b*‐values with q‐space trajectory imaging (QTI) analysis, in the human heart in vivo.

**Methods:**

Ten healthy volunteers were scanned on a 3T scanner. We designed time‐optimal gradient waveforms for tensor‐valued diffusion encoding (linear and planar) with second‐order motion compensation. Data were analyzed with QTI. Normal values and repeatability were investigated for the mean diffusivity (MD), fractional anisotropy (FA), microscopic FA (μFA), isotropic, anisotropic and total mean kurtosis (MKi, MKa, and MKt), and orientation coherence (*C*_c_). A phantom, consisting of two fiber blocks at adjustable angles, was used to evaluate sensitivity of parameters to orientation dispersion and diffusion time.

**Results:**

QTI data in the left ventricular myocardium were MD = 1.62 ± 0.07 μm^2^/ms, FA = 0.31 ± 0.03, μFA = 0.43 ± 0.07, MKa = 0.20 ± 0.07, MKi = 0.13 ± 0.03, MKt = 0.33 ± 0.09, and C_c_ = 0.56 ± 0.22 (mean ± SD across subjects). Phantom experiments showed that FA depends on orientation dispersion, whereas μFA was insensitive to this effect.

**Conclusion:**

We demonstrated the first tensor‐valued diffusion encoding and QTI analysis in the heart in vivo, along with first measurements of myocardial μFA, MKi, MKa, and *C*_c_. The methodology is technically feasible and provides promising novel biomarkers for myocardial tissue characterization.

## Abstract 2

Click here for author‐reader discussions

## 1 INTRODUCTION

Cardiac diffusion MRI (dMRI) is an emerging method for noninvasive characterization of the myocardium without the need for contrast agents. Currently, the most common dMRI method is DTI.^1^ It has been applied in a range of pathologies including hypertrophic cardiomyopathy,^2^, ^3^ dilated cardiomyopathy,^4^ infarction^5^ and amyloidosis,^6^ and remodeling following aortic stenosis^7^; the typical hallmark of pathology in the myocardium is an increase in mean diffusivity (MD) and decrease in fractional anisotropy (FA). DTI characterizes the diffusion process using a single diffusion tensor that represents the average diffusion features of tissue in each imaging voxel. Therefore, it cannot account for non‐Gaussian diffusion that may result from restrictions, disordered structural anisotropy, or tissues with heterogeneous density.^8^, ^9^ Furthermore, it has poor sensitivity and specificity whenever tissue is heterogeneous or complex, leading to a limited capability to detect and distinguish processes that involve multiple cell populations with different orientations and features.^10^, ^11^

The presence of non‐Gaussian diffusion in the myocardium has been demonstrated in preclinical investigations, such as in fixed mouse, pig,^12^ rat,^13^, ^14^ dog,^15^ and rabbit hearts.^16^ More sophisticated signal models can more accurately describe the non‐Gaussian diffusion observed in tissue. Proposed methods include the stretched exponential model in ex vivo rat hearts,^17^ and diffusion kurtosis imaging (DKI) in ex vivo porcine hearts.^18^ McClymont et al. systematically compared various signal models, including stretched exponential, DKI, bi‐exponential, truncated Gaussian, gamma and beta models, and showed that in the high b‐value regime (> 2 ms/μm^2^) the beta distribution model fit best to the data.^14^ It was observed that, unlike MD, diffusion kurtosis and skewness along the second and third eigenvectors of the diffusion tensor were able to discriminate sham from hypertrophic hearts, offering potential biomarkers of cardiac microstructure.

Measuring the effects of microscopic anisotropy and multi‐Gaussian diffusion offers the potential for greater specificity than the methods described previously. This can be achieved by performing diffusion encoding at high *b*‐values along multiple directions per shot, such as by using double diffusion encoding^19^ or arbitrary gradient waveforms.^20^, ^21^ This approach is often called “tensor‐valued diffusion encoding,” as the diffusion encoding is no longer fully described by a vector (direction and magnitude) but rather requires a tensor to also capture its shape. For example, Lasič et al. proposed a combination of linear and spherical b‐tensor encoding that could probe the microscopic FA (μFA), which describes the diffusion anisotropy without being confounded by orientation dispersion.^20^ Moreover, the relation between μFA and FA reflects the degree of orientation dispersion that could also inform on cardiac health and disease.^20^ In particular, the microscopic orientation coherence (*C*_c_) can be used to as a descriptor of the coherence of the underlying microscopic structures.^21^ Several preclinical studies that go beyond DTI have been performed to examine the heart ex vivo. These include double diffusion encoding for measuring μFA,^22^ oscillating gradients for examining time‐dependent diffusion,^23^ non‐Gaussian signal models for quantification of diffusion kurtosis,^14^ compartmental models,^24^ time‐dependent diffusion for estimation of biophysical properties,^12^, ^25^ and tensor‐valued encoding^26^ for assessing microscopic diffusion anisotropy.

Tensor‐valued diffusion encoding, in particular, can be a highly efficient probe of microscopic anisotropy and multi‐Gaussian diffusion, and has found application in various organs in brain and body imaging.^27^, ^28^, ^29^, ^30^, ^31^, ^32^ By using numerical optimization of the gradient waveforms,^33^, ^34^ b‐tensors of arbitrary shape and high *b*‐value per unit time can be produced, such as linear, planar, and spherical b‐tensor encoding (LTE, PTE, and STE), and analyzed using the q‐space trajectory imaging (QTI) framework.^21^ By performing diffusion weighting with multiple b‐tensor shapes, parameters such as MD, FA, μFA, *C*_c_, as well as the isotropic, anisotropic, and total mean kurtosis (MKi, MKa, and MKt) can be estimated.^9^, ^20^, ^21^

Given the proposed approach, we argue that cardiac QTI will likely enable higher specificity in diffusion MRI measurements than DTI and DKI. Previously, MK was shown to be better than DTI in distinguishing hypertrophic from normal myocardium in an ex vivo rat model.^14^ Decomposing MK into anisotropic and isotropic components using QTI may provide greater insight into the underlying microstructural changes in hypertrophy. FA is influenced by diffusion anisotropy and orientation dispersion, both of which are ubiquitous in the heart and cannot be distinguished by FA alone. QTI provides the opportunity to disentangle these two features, with μFA and $C_{C}$ providing separate measures of diffusion anisotropy and orientation dispersion, respectively.

Distinguishing anisotropy and orientation dispersion is relevant in myocardium where heterogeneous micro‐environments, intravoxel rotation of cardiomyocytes, and branching of cardiomyocytes are routinely observed, and to which current DTI methods have poor specificity. For example, heterogeneous micro‐environments may be found at the subepicardial and subendocardial borders where voxels contain both cardiomyocytes and blood, as well as at interfaces of normal and diseased myocardium. There is a transition in cardiomyocyte orientation from left to right‐handed orientation that manifests as a transmural variation in helix angle, and a degree of intravoxel rotation of cardiomyocytes that depends on the voxel size.^35^ There are instances of branching and multiple population of cardiomyocytes at the right‐ventricle insertion points. Furthermore, random cardiomyocyte orientation can be observed in instances of cardiomyocyte disarray in disease such as hypertophic cardiomyopathy.^2^

**FIGURE 1:**
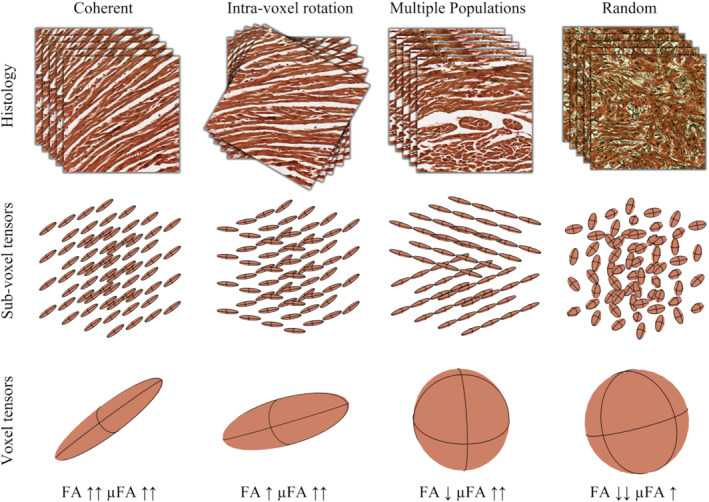
Schematic microscopic (subvoxel) and macroscopic (voxel) tensors corresponding to underlying histology illustrated by layers of (left to right) coherently organized cardiomyocytes (e.g., in healthy myocardium), dispersed cardiomyocytes (e.g., in the presence of intravoxel rotation); multiple populations of cardiomyocytes (e.g., at the right ventricle insertion points); and randomly organized cardiomyocytes (e.g., in cardiomyocyte disarray). Histology slide (random configuration) adapted from Nichols et al.^36^ FA, fractional anisotropy.

To illustrate the added information from QTI, we demonstrate the correspondence among macroscopic (voxel) tensors derived from DTI, microscopic (subvoxel) tensors derived from b‐tensor encoding, and histology in Figure 1. It shows that voxels populated by highly aligned cardiomyocytes will yield high FA and μFA. Whenever the alignment of cardiomyocytes is reduced, such as multiple cardiomyocyte populations with distinct orientations, the FA is reduced, whereas μFA is unchanged. Finally, if the cardiomyocyte orientations are random, the FA tends toward zero, whereas the μFA is still high. Thereby, the effect of actual loss of diffusion anisotropy can be disentangled from the effect of orientation coherence.

Although greater specificity for cardiac dMRI is sought, there are considerable challenges associated with the use of both conventional and tensor‐valued diffusion encoding at moderate to high *b*‐values in the beating heart. Chief among them are cardiac and respiratory motion, short transversal relaxation times (*T*_2_), and the need for higher *b*‐values than required for DTI.^8^, ^20^, ^37^ Furthermore, nonlinear b‐tensor encoding (e.g., PTE and STE) generally has a lower efficiency than conventional LTE, meaning that it requires slightly longer encoding times to reach a given *b*‐value.^34^, ^38^ It is likely that these challenges have stymied the in vivo application of non‐Gaussian diffusion MRI in the heart. To meet these challenges, we previously developed techniques for tensor‐valued diffusion encoding with motion compensation to arbitrary order, and we used these techniques at relatively low *b*‐values in heart in vivo.^39^, ^40^ In the work by Szczepankiewicz et al., we developed numerical gradient waveform optimization to achieve efficient encoding with arbitrary b‐tensor shapes while simultaneously ensuring motion compensation and negligible effects from concomitant gradients, thereby enabling an experimental design that is compatible with clinical scanners and scan times.^40^ In this work, for the first time, we demonstrate tensor‐valued diffusion encoding at high *b*‐values^41^ along with a QTI analysis in the human heart in vivo. We validate our measurements using a novel multicompartment phantom and examine baseline parameter values and parameter repeatability in a cohort of healthy volunteers.

## 2 THEORY

QTI is a theoretical framework that can probe the diffusion process and underlying tissue microstructure based on tensor‐valued diffusion encoding.^21^ It can be considered an extension of DKI,^8^ as it estimates the covariance tensor, which also contains the kurtosis tensor. Importantly, tensor‐valued diffusion encoding involves the use of multiple *b*‐values and b‐tensors with various shapes, rather than diffusion weighting in a single direction as applied in conventional diffusion encoding. This facilitates the separation of microscopic diffusion anisotropy from heterogeneous isotropic diffusivities,^9^, ^10^, ^20^, ^21^ which is not possible with conventional diffusion encoding. The analysis yields a set of DTI, DKI and QTI parameters, of which we will use the MD, FA, μFA, MKi, MKa, MKt, and *C*_c_.

Here, we give an overview of the basic theory of QTI and its relation to tensor‐valued diffusion encoding, but we encourage the reader to pursue a more detailed description in the work by Westin et al.^21^ Briefly, we assume that the tissue in each voxel can be approximated by a distribution of diffusion tensors, where each diffusion tensor represents a part of the tissue where the diffusion is Gaussian and nonexchanging. In this multi‐Gaussian system, a truncated cumulant expansion can be performed, to yield the following signal representation^21^:

(1) $$S\left(B\right)\approx S_{0}\;\exp\left(-B:D+\frac{1}{2}B^{⨂2}:\mathbb{C}\right)$$

where B is the b‐tensor; D is the average diffusion tensor; $B^{⨂2}$ is the outer product of the b‐tensor with itself; ℂ is the fourth‐order diffusion covariance tensor; and “:” denotes the double inner product. Parameters such as MD and FA are computed from the diffusion tensor, whereas the covariance tensor is needed to calculate the μFA, MKi, and MKa. To highlight how the MK parameters affect the signal, we may simplify this expression by considering the powder‐averaged signal (arithmetic average over directions). Then, Equation (1) can be written in terms of the conventional *b*‐value and the b‐tensor shape ($b_{\Delta }$), according to Refs. 9, 27 and 34:

(2) $$S\left(b\right)\approx S_{0}\;\exp\left(-b\mathrm{MD}+\frac{1}{6}b^{2}MD^{2}\left({\mathrm{MKi}+b}_{\Delta }^{2}\mathrm{MKa}\right)\right).$$

This expression shows how heterogeneous isotropic diffusivities, MKi, and microscopic diffusion anisotropy, MKa, both contribute to the *b* ^2^ term, and that the contribution from the latter is modulated by the shape of the b‐tensor. Finally, we note that the total kurtosis can be calculated according to MKt = MKi + MKa.^9^, ^20^

## 3 METHODS

We first sought to demonstrate the effects of orientation dispersion on DTI and QTI measurements in a reference phantom with known microstructural properties, as a form of validation. We then optimized the gradient encoding waveforms for in vivo cardiac QTI and applied these in a cohort of healthy volunteers to obtain baseline measurements and repeatability of QTI parameters.

### 3.1 Fiber Phantom Experiments

To demonstrate the contrast between FA and μFA, a fiber phantom with variable orientation dispersion was designed and fabricated. Hollow fibers were prepared using co‐electrospinning of polycaprolactone shell solution and polyethylene oxide core solution.^42^ The introduction of the surfactant polysiloxane into the polycaprolactone fibers improved their surface wettability, permitting the use of water as a diffusate for improved safety relative to a previous iteration using cyclohexane, and matching of physiological properties.^43^ Fiber strips were cut into squares with sectional areas of approximately 1.0 cm,^2^ immersed in water, and sonicated to remove air bubbles. A custom holder with two mating hollow cavities for the fibers was designed and 3D printed. The fiber squares were layered and filled the cavities to form two fiber blocks. The phantom was sealed in a glass tube with an outer diameter of 20 mm. Rotation of the two mating fiber blocks created a region at the interface such that an MRI slice can cover both regions; therefore, voxels in the regions contain two fiber populations with user controllable directions. All preparations were performed while submerged in water to minimize introduction of air bubbles.

**FIGURE 2:**
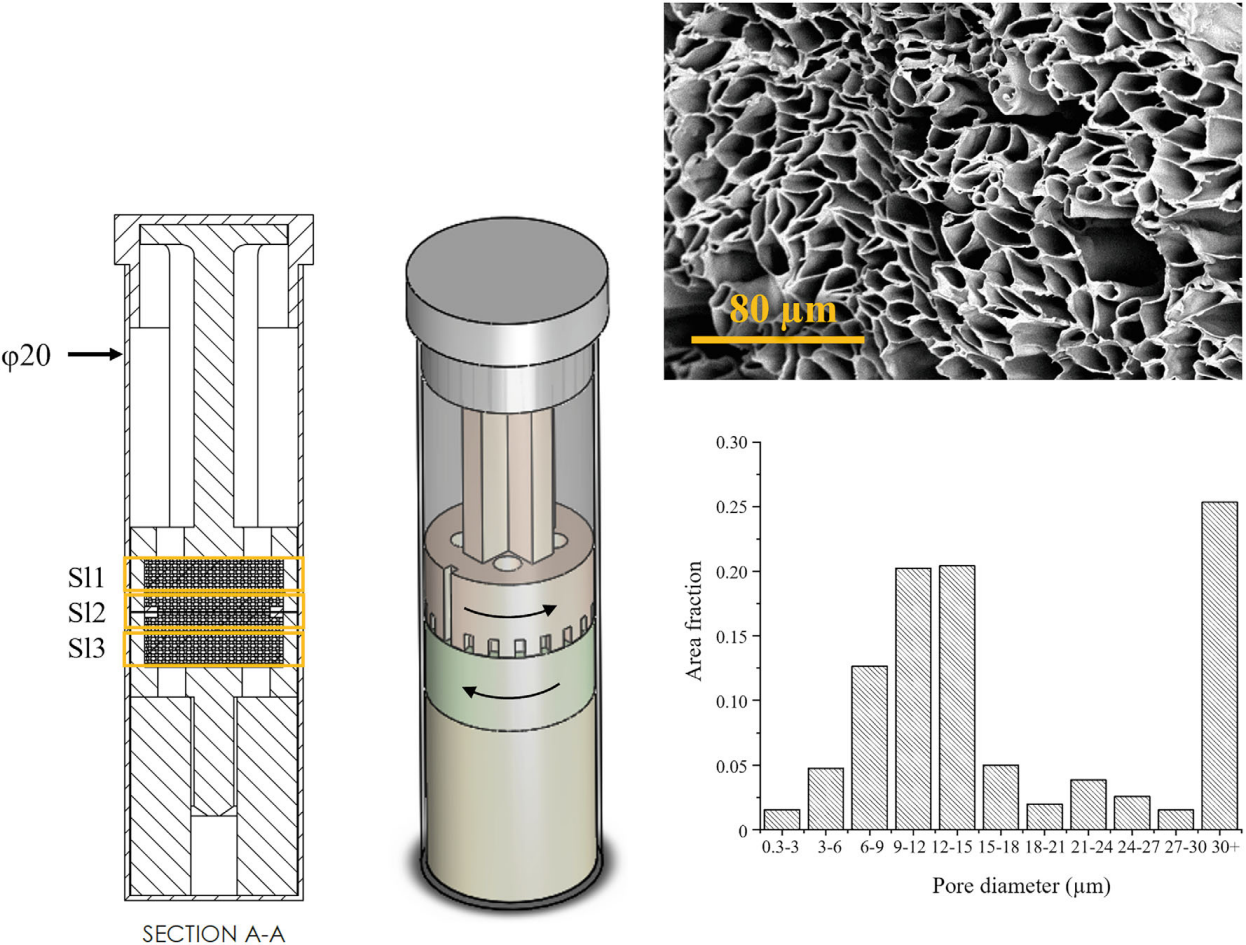
(Left) Vertical cross section and 3D view of phantom consisting of two blocks of hollow fibers that are rotated from interblock angle = 0° (parallel; as shown) to 90° (perpendicular) with respect to one another (black arrows). Slice locations 1–3 are shown in yellow. (Top right) Scanning electron microscopy image of fiber cross section at ×1000 magnification. (Bottom right) Histogram of pore diameters with 3‐μm bins up to 30 μm. All pores with diameters greater than 30 μm were grouped in a single bin, which accounts for the high area fraction.

The phantom is depicted in isometric and cross‐sectional views (Figure 2). A representative scanning electron microscopy image taken from a sample of the same fiber strip that was used to make a phantom is shown. A densely packed microstructure with irregular hollow cross‐sections was observed. The pore diameters were measured from three scanning electron microscopy images acquired from different regions of the fiber strip, and the histogram analysis indicates a preponderance of fibers with pore diameters ranging from 9 to 15 μm.

Data were acquired on a Biospec 7T MRI scanner (Bruker BioSpin MRI, Ettlingen, Germany) with a 1.5 T/m gradient system with rise time < 100 μs. Multishot 2D diffusion‐weighted spin‐echo EPI data were acquired with a product DTI sequence, and a custom free waveform sequence^44^ available at https://osf.io/t9vqn, which enables tensor‐valued diffusion encoding. Imaging was performed with FOV = 20 × 20 mm,^2^ matrix = 64 × 64, slice thickness = 3 mm, number of shots = 4, number of slices = 3, number of diffusion‐encoding directions = 15, bandwidth = 150 kHz, nontriggered, and *G*_max_ was adjusted to achieve required *b*‐values. A higher resolution was used compared with the in vivo study to exclude partial‐volume effects in the phantom. The DTI sequence parameters were TR = 3000 ms, TE = 60 ms, diffusion duration (*δ*) = 1.7 ms, time between onset of gradient pulses (Δ) = [5, 10, 20, 30, 40] ms, *b* = 1.0 ms/μm^2^, number of non‐diffusion‐weighted images = 2, and acquisition time = 20.5 min. The QTI sequence parameters were TR = 4000 ms, TE = 60 ms, diffusion‐encoding waveform duration = 20 ms, *b* = [0, 0.1, 0.5, 1.0, 2.0] ms/μm^2^, and acquisition time = 40 min. The gradient waveform was configured to yield spherical b‐tensor encoding, using the q‐space magic angle spinning (qMAS) design,^45^ repeated identically before and after the refocusing pulse. Additionally, a linear b‐tensor encoding waveform was extracted from a single axis of the qMAS waveform. The waveforms were characterized by the mean frequency (in hertz) of the power spectrum of the dephasing vector $q\left(t\right)=\gamma \int_{0}^{t}g\left(t^{\prime }\right)dt^{\prime }$.^46^

The conventional data were used to estimate diffusion tensors, MD, and FA by nonlinear least‐squares fitting with code available at https://github.com/vigente/gerardus. The tensor‐valued encoding data were used to estimate MD, FA, and μFA by linear fitting of the QTI^21^ signal representation, as implemented in the multidimensional diffusion MRI framework available at https://github.com/markus‐nilsson/md‐dmri.^47^ A central circular mask with radius of 5 voxels was defined in the central slice. This mask was propagated across slices and image volumes with different interblock rotations. MD, FA, and μFA were averaged across each region of interest (ROI), and mean values were reported for each slice and interblock angle.

### 3.2 Healthy Volunteer Experiments

To investigate baseline QTI values in the clinical setting, data were acquired in 10 healthy volunteers (age = 22 ± 3 years, 3 male and 7 female) on a Prisma 3T MRI scanner (Siemens Healthineers, Erlangen, Germany). The average heart rate for study participants was 70.4 ± 8.6 beats per minute (mean ± SD across 9 subjects). Volunteers provided written consent, and the study was performed following ethical approval.

Tensor‐valued diffusion encoding was performed with a prototype single‐shot spin‐echo sequence (version 1.24; https://github.com/filip‐szczepankiewicz/fwf_seq_resources)^48^ with EPI readout and reduced FOV imaging. The scan was cardiac‐triggered, and the subject was scanned free‐breathing without respiratory gating. The QTI sequence parameters were linear and planar b‐tensor encoding (waveform design is described subsequently); TR = 5 RR‐intervals; TE = 118 ms; resolution = 3.5 × 3.5 × 10 mm^3^ in 5 slices; FOV = 320 × 111 mm^2^; *b* = [0.1, 0.4, 0.7, 1.1, 1.5] ms/μm^2^ for [10, 20, 30, 40, 50] rotations, respectively; partial Fourier = 7/8; bandwidth = 1510 Hz/px; trigger delay ˜ 10% of end systole (ms), where end systole (ms) was measured based on cine MRI data; and total acquisition time = 25 min based on a heart rate of 60 beats per minute. The resolution was lower than in typical cardiac DTI experiments, to compensate for lower SNR caused by the higher b‐values used. Partial volume with unsuppressed blood may lead to signal contribution from blood at low *b*‐values, and this effect may be enhanced in the presence of motion compensation. We therefore used a nonzero low *b*‐value (*b* = 0.1 ms/μm^2^) to mitigate the signal contribution of flow and perfusion. The non‐self‐balanced waveform design (i.e., relatively high **q**[*t*] at the time of the refocusing pulse) meant that crushers were not needed for any of the used *b*‐values. Therefore, crushers were always turned off and did not degrade the motion compensation as observed in previous work using self‐balanced gradients.^39^ The acquisition order of *b* and diffusion‐encoding directions were pseudo‐randomized to minimize the duty cycle and avoid signal drift effects.^49^ Data were acquired during systole. The QTI experiment was repeated in the same session to investigate repeatability (*N* = 9). ECG data were exported from the scanner (*N* = 9). Cardiac triggers were extracted using open‐source software *TAPAS*,^50^ and average heart rate was calculated across the scan session in each subject.

Data postprocessing included denoising^51^ and Gibbs ringing removal^52^ performed in *MRtrix*,^53^ as well as motion and eddy current correction with extrapolated reference images^54^ performed in *Elastix*.^55^ Distortions due to heterogeneous B_0_ were not performed. All acquired images were used in the reconstruction. QTI parameters were estimated using the same approach as for the phantom experiments. Four ROIs in the septal, anterior, inferior, and lateral wall of the left ventricle in one mid‐myocardial short‐axis slice were manually defined. Parameter maps, including MD, FA, μFA, MKa, Mki and MKt, were reported in each region as mean ± SD across subjects. Repeatability in the parameter maps was assessed by Bland–Altman analysis,^56^ and mean ± 1.96 SD was reported across subjects and regions.

To detect potential effects of time‐dependent diffusion, DTI parameters based on two waveforms for LTE were compared. The two waveforms were derived from the PTE waveform and selected to represent the longest and shortest diffusion times, denoted as LTE (long t_d_) and LTE (short t_d_), respectively (see Section 3.3). All other acquisition parameters were identical as described previously. The highest *b*‐value was 0.7 ms/μm^2^, bounded by the low efficiency of the LTE (short t_d_) waveform, and the lowest b‐value was 0.4 ms/μm^2^ to mitigate the effects of perfusion and to maximize the effects of restrictions. DTI signal representations were fitted separately to LTE (long t_d_) and LTE (short t_d_) data sets using nonlinear least‐squares fitting. Average MD and FA were calculated in the four ROIs. A two‐sample t‐test was applied to test the null hypothesis that the MD and FA had equal means, using a significance threshold of 0.05. The parameter distributions and averages of MD and FA were plotted using data pooled across ROIs and subjects (*N* = 9). At longer diffusion‐encoding times, water molecules can travel farther and sense more restrictions to diffusion, such as cell membranes and organelles, thereby reducing the apparent diffusivity. As cardiomyocytes are highly anisotropic in shape, there is a greater tendency for water molecules to sense restrictions along the short axis of the cell rather than its long axis. This leads to relatively low apparent diffusivity and potential diffusion‐time dependency along the short axis of the cardiomyocyte. We expect that effects of diffusion time and restriction will manifest as a lower MD and higher FA when using LTE (long t_d_) as compared with LTE (short t_d_).^23^

### 3.3 Gradient waveform design for in vivo cardiac QTI measurements

Gradient waveforms were generated in the open‐source optimization framework by Sjölund et al.^34^ found at https://github.com/jsjol/NOW, using the extensions for concomitant gradient compensation^33^ and moment nulling.^40^ All waveforms were motion‐compensated up to second order (position, velocity, and acceleration) by imposing that the magnitude of the motion‐encoding vector of order *n*, $m_{n}=\gamma \int_{0}^{\mathrm{TE}}t^{n}g\left(t\right)dt$, is equal to zero.^40^ The encoding times were 43.4 ms before and after the refocusing pulse, and the waveforms were allowed to be asymmetric to boost the encoding efficiency.

To minimize the required encoding time, and therefore achieve minimal TE and maximal SNR, we performed a combination of linear and planar b‐tensor encoding as opposed to combining linear with spherical b‐tensor encoding. To avoid peripheral nerve stimulation (PNS), the maximal slew rate of the gradient waveforms was limited to 100 T/m/s, and the PNS level was predicted using the SAFE model^57^ implemented in *MATLAB* (https://github.com/filip‐szczepankiewicz/safe_pns_prediction).^38^ The maximum predicted magnitude of PNS based on the scanner limits was 72%, 21%, and 51% for PTE, LTE, (long t_d_) and LTE (short t_d_), respectively. To achieve diffusion‐time matching between LTE and PTE, without compromising the encoding efficiency of the waveform, the LTE waveform was defined from the PTE waveform. Briefly, this is done by selecting the waveform that points along the eigenvector of the largest eigenvalue of $\int_{0}^{\mathrm{TE}}g\left(t\right)\cdot g\left(t\right)dt$, where g(t) is the effective gradient waveform.^58^ This ensures that the LTE waveform has identical diffusion‐time characteristics to the PTE along at least one direction, while not suffering from poor encoding efficiency. We denote this encoding as LTE (long t_d_). Additionally, to cover the full range of diffusion‐time dynamics of the PTE, we created an LTE waveform that had a short diffusion time by selecting the waveform that is orthogonal to the first. We denote this encoding as LTE (short td).

**FIGURE 3:**
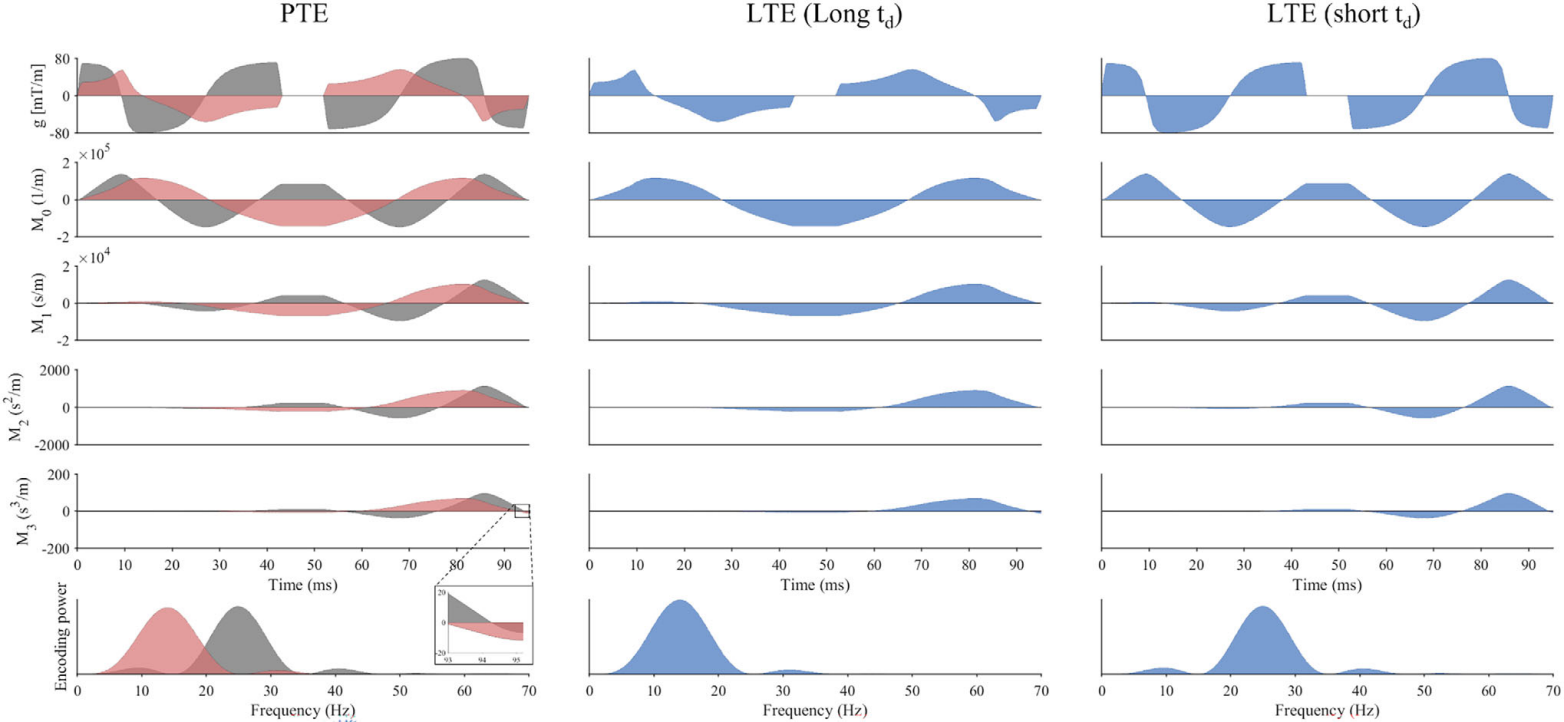
(Top to bottom) Effective diffusion‐encoding gradient waveforms for b‐tensor encoding, zeroth to third‐order gradient moments; and encoding power spectrum for (left to right) planar b‐tensor encoding (PTE), linear b‐tensor encoding (LTE) (long t_d_), and LTE (short t_d_) corresponding to intermediate, low, and high mean frequencies, respectively. Waveforms were compensated for up to second‐order motion, and residual M_3_ can be seen at the end of the waveforms (see inset). Encoding power spectrum (in hertz) is defined as the Fourier transform of **q**(t).^38^ There is a clear distinction in the encoding power spectrum between LTE (long t_d_) and LTE (short t_d_), while PTE contains contributions from both LTE waveforms. The three colors (red, gray, and blue) correspond to orthogonal directions. Other sequence features were omitted for clarity. Waveforms were generated using a modified NOW toolbox.^34^

The waveforms for PTE, LTE (long t_d_), and LTE (short t_d_) are shown in Figure 3. The two LTE waveforms are based on the two orthogonal waveforms that constitute the PTE waveform. The nominal trajectories of zeroth to second‐order moments terminate effectively at zero, meaning that the waveforms are motion‐compensated up to acceleration, whereas the third‐order moments are not nulled. However, due to interpolation and limited numerical precision at the scanner, the first and second‐order moments are nonnegligible in practice. The magnitude of the first, second, and third‐order motion encoding from the diffusion‐encoding waveforms were 6.8 × 10^−1^ s/m, 7.8 × 10^−2^ s^2^/m, 13 s^3^/m (PTE; *b* = 1.5 ms/μm^2^); 8.3 × 10^−1^ s/m, 9.7 × 10^−2^ s^2^/m, 16 s^3^/m (LTE long t_d_; *b* = 1.5 ms/μm^2^); and 3.3 × 10^−1^ s/m, 3.8 × 10^−2^ s^2^/m, 6.5 s^3^/m (LTE short t_d_; *b* = 0.7 ms/μm^2^); respectively. These moments can be equally expressed as magnetic field gradient moments in units of *T*s^(*n*+1)^/m by dividing by the proton gyromagnetic ratio. Figure 3 also shows orthogonal projections of the encoding power spectrum of the dephasing vector **q** (t), where the peaks appear at 14 Hz for LTE (long t_d_), 25 Hz for LTE (short t_d_), and a combination of the two frequencies for the PTE waveform. Other constraints include enforcing the L2‐norm of the gradient amplitude as a function of time (< 80 mT/m), slew rate (< 100 T/m/s), b‐tensor shape (Frobenius distance error < 0.5%), and concomitant gradient balance (Maxwell index < 100 [mT/m]^2^ ms).

## 4 RESULTS

**FIGURE 4:**
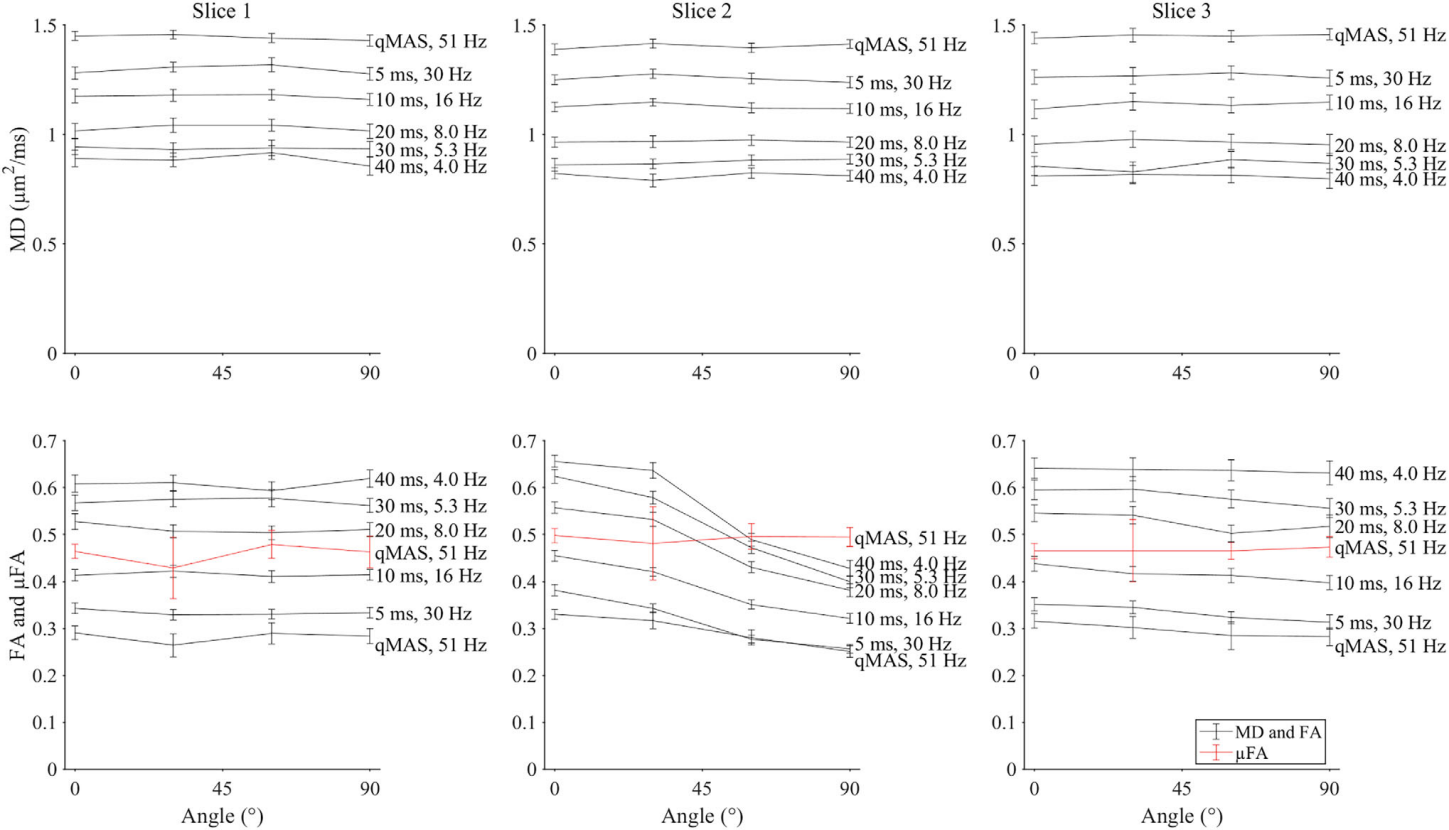
Dependence of mean diffusivity (MD; black lines), FA (black lines), and microscopic FA (μFA; red lines) on interblock angle and (left to right) slice positions 1 to 3 (mean ± SD across voxels). Diffusion times (ms) given for pulsed gradient spin‐echo (PGSE) experiments, and mean frequencies of the power spectrum of **q**(t) given for PGSE and q‐space magic angle spinning (qMAS) experiments. MD increases and FA decreases as the diffusion time is decreased. FA decreases with increasing interblock angle in Slice 2, simulating increased fiber orientation dispersion. In contrast, μFA remains relatively insensitive to interblock angle, highlighting the strength of μFA as a metric of anisotropy that is not biased by orientation dispersion.

The DTI data in the phantom demonstrated time‐dependent diffusion across all slices, where shorter diffusion times (higher mean frequencies) yielded higher MD and lower FA (Figure 4; Figures S1 and S2). The qMAS‐derived LTE data had the highest mean frequency of 51 Hz, and generally the highest MD and lowest FA. In Slices 1 and 3, fibers were always fully aligned, and MD and FA were insensitive to interblock angle. In Slice 2, an increasing interblock angle caused the FA to decrease, while MD was unaffected. Finally, the μFA was the same across all slices and remained approximately constant for all interblock angles (i.e., it was unaffected by the subvoxel distribution of fiber directions).

**FIGURE 5:**
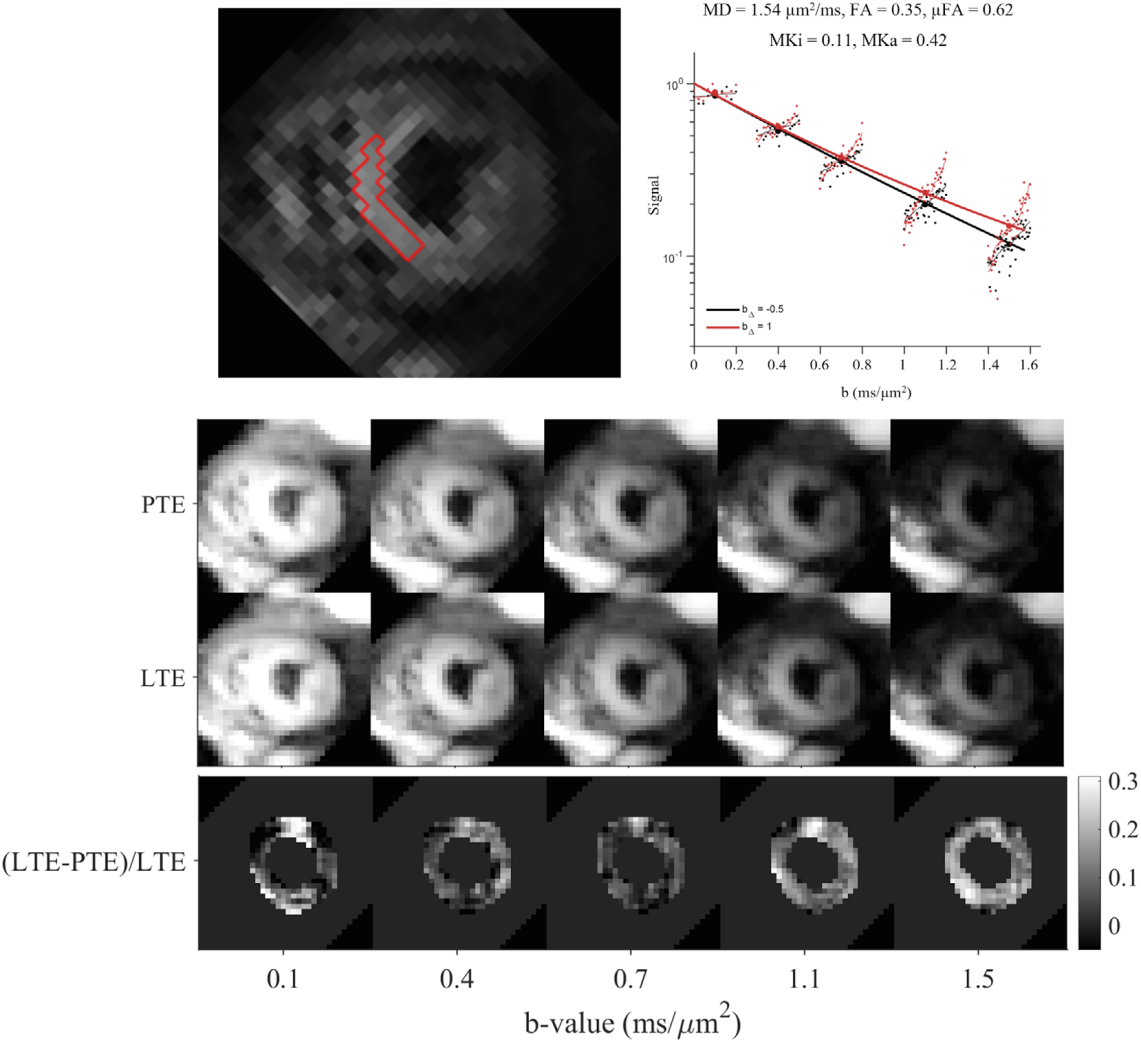
(Top) Normalized signal‐attenuation curves showing divergence between linear (red; *b*_Δ_ = 1) and planar (black; *b*_Δ_ = −0.5) tensor encoding, particularly at higher b‐values. Data are reported in an example region of interest (ROI) in the myocardium of 1 healthy volunteer. Signals were averaged across the ROI and used to fit a covariance model. Data were acquired in five shells and plotted with offsets in b for better visualization of data and fitting. (Middle) Powder average of PTE and LTE log signal images, sorted by *b*‐value. (Bottom) Relative difference images between PTE and LTE show that contrast generally increases with *b*‐value. MKa, anisotropic mean kurtosis; MKi, isotropic mean kurtosis.

Cardiac QTI measurements were completed successfully in all 10 volunteers. An example of a midventricular short‐axis slice is shown alongside the signal attenuation across *b*‐values and diffusion‐encoding directions in an ROI in the septal wall (Figure 5). At low *b*‐values, *b* < 0.5 ms/μm^2^, the signal from LTE and PTE is similar, as expected from theory. However, as the b‐value increases, the contrast between PTE and LTE becomes increasingly pronounced; this is the hallmark of microscopic diffusion anisotropy.^10^, ^20^, ^59^ Images of the logarithm of the powder‐averaged signal when using PTE and LTE illustrate the diffusion contrast and overall image quality. The relative difference between PTE and LTE generally increases with b‐value, corresponding to the divergent signal attenuation curves, which is a hallmark of microscopic diffusion anisotropy.^10^, ^20^ However, there were regions in the inferior wall at *b* = 0.1 ms/μm^2^ with large differences that may be artefactual.

**FIGURE 6:**
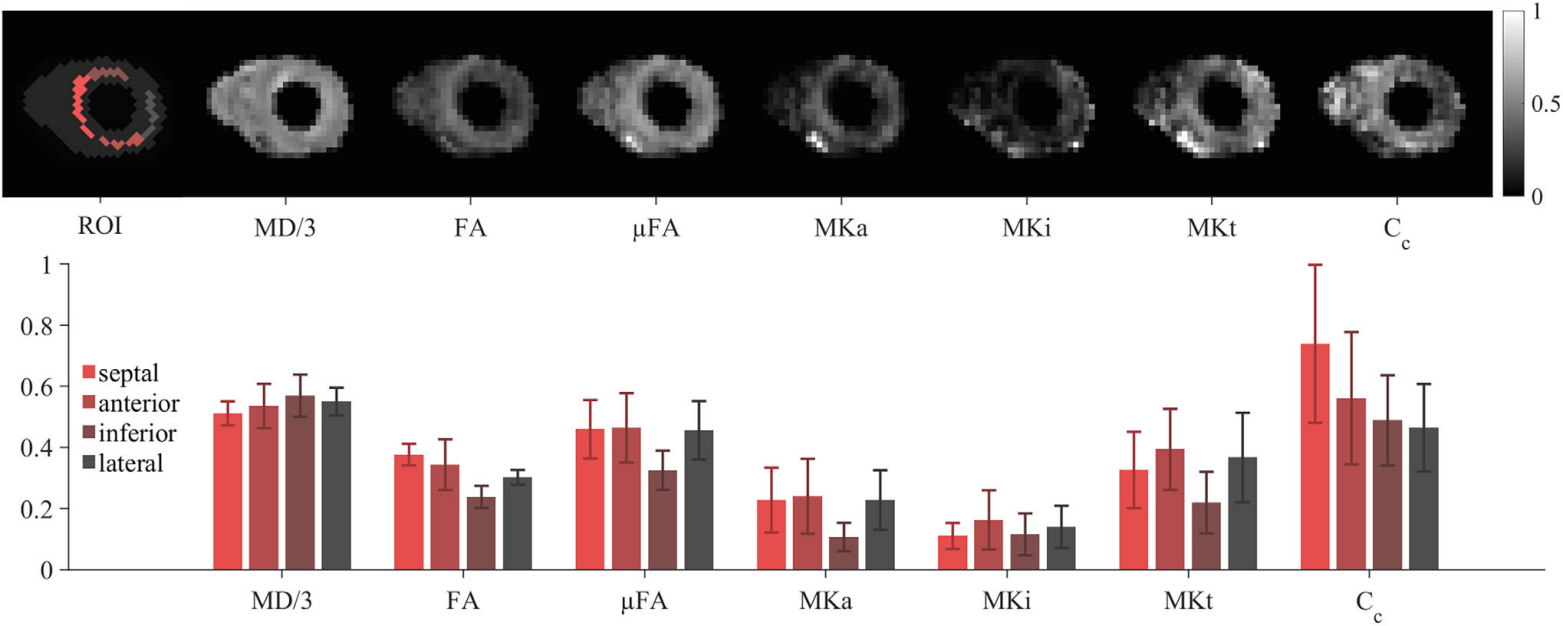
(Top) Example cardiac q‐space trajectory imaging (cQTI) maps and (bottom) values in a middle short‐axis slice in the left‐ventricular (LV) wall (*N* = 10). MD (in μm^2^/ms), FA, μFA, MKa, MKi, total mean kurtosis (MKt), and microscopic orientation coherence (*C*_c_) shown. Mean ± SD are reported across subjects. The measurements were more consistent across the septal, anterior, and lateral walls. In the inferior wall, higher MD and lower FA, μFA, and MKa were observed, which may be associated with susceptibility effects near the posterior vein.

Example QTI parameter maps are depicted in Figure 6 alongside average values in four ROIs in the left‐ventricular myocardium. These baseline values in healthy volunteers are summarized in Table 1. Overall, regions with higher MD corresponded to regions with lower FA and vice versa. μFA was uniformly higher than FA. Measures of FA, μFA, MKa, and MKt were lower in the inferior wall compared with other regions.

**TABLE 1:** Cardiac q‐space trajectory imaging parameter values in a middle short‐axis slice in the left‐ventricular wall (*N* = 10). Mean ± SD across subjects.

Parameters	Septal	Anterior	Inferior	Lateral	Average
MD (μm^2^/ms)	1.53 ± 0.12	1.61 ± 0.22	1.71 ± 0.21	1.65 ± 0.14	1.62 ± 0.07
FA	0.38 ± 0.04	0.34 ± 0.08	0.23 ± 0.04	0.30 ± 0.02	0.31 ± 0.03
μFA	0.46 ± 0.10	0.46 ± 0.11	0.33 ± 0.06	0.46 ± 0.10	0.43 ± 0.07
MKa	0.23 ± 0.11	0.24 ± 0.12	0.11 ± 0.05	0.23 ± 0.10	0.20 ± 0.07
MKi	0.11 ± 0.04	0.16 ± 0.10	0.12 ± 0.07	0.14 ± 0.07	0.13 ± 0.03
MKt	0.33 ± 0.12	0.39 ± 0.13	0.22 ± 0.10	0.37 ± 0.15	0.33 ± 0.09
*C*_c_	0.74 ± 0.26	0.56 ± 0.22	0.49 ± 0.15	0.46 ± 0.14	0.56 ± 0.22

Abbreviations: *C*_c_, orientation coherence; FA, fractional anisotropy; MD, mean diffusivity; MKa, anisotropic mean kurtosis; MKi, isotropic mean kurtosis; MKt, total mean kurtosis; μFA, microscopic fractional anisotropy.

**FIGURE 7:**
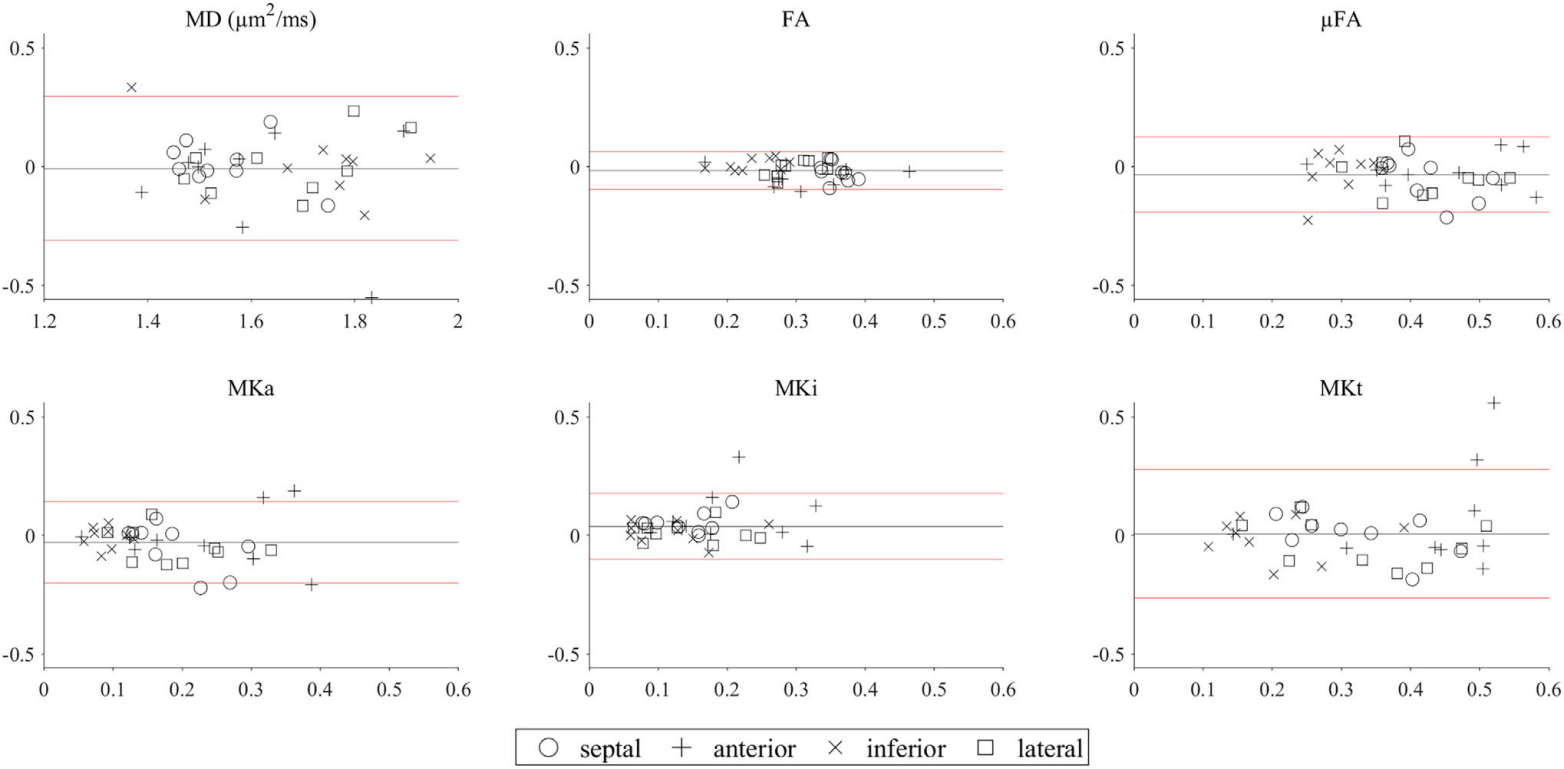
Bland–Altman plots showing agreement in cQTI between Scans 1 and 2 within ROIs. Mean difference ± 1.96 SD are given by black and red lines, respectively (*N* = 9). These data can serve as a benchmark for future improvement, and can be valuable when planning future studies.

Bland–Altman plots illustrate an overall good repeatability based on test–retest data and QTI analysis (Figure 7). The test–retest difference in QTI parameters (mean ± 1.96 SD across subjects and ROIs) were ΔMD = −0.01 ± 0.31 μm^2^/ms, ΔFA = −0.02 ± 0.08, ΔμFA = −0.03 ± 0.16, ΔMKa = −0.03 ± 0.17, ΔMKi = 0.04 ± 0.14, and ΔMKt = 0.01 ± 0.27.

**FIGURE 8:**
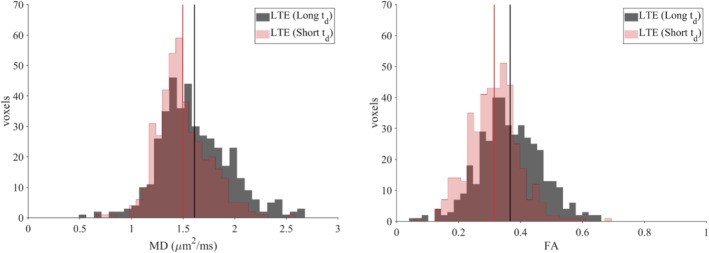
MD and FA histograms for LTE (Long t_d_) and LTE (Short t_d_). Mean MD = [1.61, 1.50] μm^2^/ms and mean FA = [0.37, 0.32], respectively (vertical lines). Data were pooled across subjects (*N* = 9) and ROIs.

Histograms of MD and FA for LTE (long t_d_) and LTE (short t_d_) are presented in Figure 8. Both the mean MD and mean FA were higher in the former, with mean MD = 1.61 versus 1.50 μm^2^/ms and mean FA = 0.37 versus 0.32, respectively. Both parameters were significantly different (*p* < 1 × 10^−7^); however, in the case of MD, the direction of the effect was opposite to what is expected from an interaction between restrictions and diffusion time.

## 5 DISCUSSION

We report the first measurements of μFA and MK in the human heart in vivo. We observed that μFA > FA, which indicates the presence of intravoxel orientation dispersion^10^, ^20^, ^21^ and is consistent with the known transmural variation in cardiomyocyte orientation. Both μFA and MK were lower in the myocardium than in brain white matter, where they have been reported as 0.74 and 0.93, respectively.^21^ This may reflect the larger cell diameter and higher permeability of cardiomyocytes relative to axons. The relatively low MKi reflects low intravoxel variation in isotropic diffusivity,^9^ which is expected for tissue with low microstructural heterogeneity in healthy myocardium.

As diffusion time effects have been observed in previous work in vivo,^39^ it is reasonable to assume that they may be present in the QTI data as well. To minimize potential bias, we implemented a version of PTE that shares the frequency content of LTE along one axis. Time‐dependent diffusion typically manifests as a reduction in MD as diffusion‐encoding times become longer.^39^ This is because as diffusion‐encoding times increase, there is a greater likelihood of water molecules encountering membranes or organelles that restrict diffusion. In this study, we observed LTE with long t_d_ had a higher MD than LTE with short t_d_, suggesting that diffusion time was not a dominant effect. This could be due to the necessarily long diffusion‐encoding waveforms in the clinical setting for cardiac QTI, leading to smaller relative differences in encoding power of LTE with short t_d_ and long t_d_. Consider also human cardiomyocytes on the scale of about 141 μm in length and 19 μm in width.^60^ Assuming hollow cylinders with MD = 1.6 μm^2^/ms, we would expect that the intermediate diffusion time (where some water molecules feel the effects of restriction) would be about 1.6 s and 28 ms, respectively. Based on the applied waveforms, we expect to be measuring primarily free diffusion along the cardiomyocyte long axis, and intermediate diffusion (i.e., between free and restricted diffusion) along the cardiomyocyte short axis. This suggests that time‐dependent diffusion effects may be more pronounced in the presence of even longer diffusion‐encoding times. Further investigation is required to identify the conditions in which diffusion time effects may have a greater impact.

There is also potential for differences in MD arising from different acquisition/postprocessing methods. In recent DTI studies, Das et al.^2^, ^61^ reported average myocardial MD of between 1.46 and 1.47 μm^2^/ms, which was 11% to 12% lower than in our data. In cardiac DTI, MD is routinely underestimated by a degree, as diffusion kurtosis is not accounted for as has been in this study, and this may help put the MD results in context.

Phantoms are an invaluable tool for validation of new methods and applications. Previous hollow fiber phantoms have been used to evaluate methods for assessing time‐dependent diffusion^62^ and μFA.^63^ Here, we designed and built a novel phantom that combines hollow fibers with pore‐size distribution more closely matching cardiomyocytes, and with a rotating mechanism for simulating fiber dispersion. The results demonstrate confirmation that, unlike FA, MD and μFA are insensitive to fiber dispersion. Furthermore, we observed that MD increased, and FA decreased, with mean frequency of the power spectrum of **q**(*t*), demonstrating clear diffusion time effects in contrast to the in vivo data. This difference may appear due to the longer diffusion times used at the clinical scanner (a consequence of the relatively low gradient system performance in human MRI systems) and may be indicative of a higher permeability in myocardium compared with the plastic used in the phantom.^64^, ^65^ Here, we were limited by the size of the phantom; therefore, a preclinical scanner with high gradient performance was used. Further development of more sophisticated phantoms, coupled with evaluation on clinical scanners, will add further insight.

Tensor‐valued encoding in the heart in vivo is challenging for several reasons. Most prominently, cardiac motion necessitates a motion‐compensated diffusion encoding; otherwise, the irregular movement of the heart causes signal dropout.^66^, ^67^ Constraining the motion encoding reduces the encoding efficiency and extends the required encoding time.^38^, ^40^ Coupled with a relatively short T_2_ compared with brain, the SNR is relatively low. Simulations of brain parenchyma suggest that there is an upward bias and lower precision in MKa, MKi, and MKt at low SNR.^48^ uFA appears to be more accurate and precise at low SNR, but a similar positive bias and lower precision are observed when the true value of uFA is low. Further investigation is needed to determine potential bias in the myocardium at varying SNR. These issues were addressed by tailoring acceleration‐nulled PTE gradient waveforms that had an efficient LTE subset,^40^ in combination with large voxels, denoising, and Gibbs‐ringing removal. Nevertheless, the encoding power spectra of the dephasing vector **q** (t) in LTE and PTE were not matched exactly, which may cause a bias in QTI parameters.^38^, ^62^, ^68^ Other considerations include the following. First, we observed potential bias in parameters (higher MD and lower FA, μFA, and MK) in the inferior wall due to susceptibility artifacts near the posterior vein. This may be ameliorated by acquiring additional data with reversed phase encoding.^69^, ^70^ Second, the slice‐selection gradients were neglected in the experimental design and analysis but are known to contribute to motion encoding.^71^ Third, the sample size was small and consisted of young healthy volunteers with relatively steady heart rates. Including a larger cohort of patients would be more representative of the clinical setting. Fourth, the acquisition time was relatively long due to the extensive sampling scheme. Improvements in scan time would be key for application of cardiac QTI in the clinic. This may be achievable by using a parsimonious acquisition protocol and adapted analysis.^27^, ^72^, ^73^, ^74^ Fifth, as with cardiac diffusion MRI in general, there may be a small number of images in which the motion compensation performs suboptimally, such as caused by severe subject motion. This effect was mitigated by extensive sampling of q‐space, with 150 q‐samples per waveform. In addition, outlier rejection methods could be used to exclude “bad” data, although such methods would require further refinement and validation. In future work, we will refine the sampling protocol and postprocessing for optimal spatial resolution to achieve acquisition times that are compatible with clinical research times. Fifth, there is a degree of slice‐to‐slice variation that is related to the heterogeneous local motion properties and changing curvature of the heart. This can, for instance, lead to motion‐induced signal loss and greater partial‐volume effects near the apex. In this study, we reported data from a single slice only. Further work will be needed to evaluate slice‐to‐slice variation and to identify strategies for its mitigation.

## 6 CONCLUSIONS

We have demonstrated, for the first time, the technical feasibility of QTI in the human heart in vivo along with normal values and a quantification of the measurement reproducibility. Despite its many challenges, the measurement was facilitated by enabling tensor‐valued diffusion encoding at high *b*‐values. We observed that μFA > FA, which indicates the presence of intravoxel orientation dispersion and the relatively low MKi, suggests a limited microstructural heterogeneity in healthy myocardium. Validation in a custom hollow‐fiber phantom showed that μFA was insensitive to orientation dispersion, and that MD and FA were sensitive to diffusion time effects. Furthermore, we found no clear evidence that restrictions and diffusion time was a confounding factor, which may be linked to higher permeability in comparison to the phantom. These first in vivo measurements of μFA, mean kurtosis, and microscopic orientation coherence highlight the potential for improved specificity in characterizing the myocardial microstructure, with exciting opportunities for application in the healthy and diseased myocardium.

## AUTHOR CONTRIBUTIONS

Study conceptualization, design and planning (Irvin Teh, Filip Szczepankiewicz), funding acquisition (Irvin Teh, Sven Plein, Erica Dall'Armellina, Jürgen E. Schneider, Nadira Y. Yuldasheva, Filip Szczepankiewicz), phantom design and build (Irvin Teh, Jordan H. Boyle, Geoff J. M. Parker, Fenglei Zhou), SEM data acquisition and analysis (Geoff J. M. Parker, Fenglei Zhou), MRI pulse sequence design (Filip Szczepankiewicz), experiment design (Irvin Teh, Filip Szczepankiewicz), MRI data acquisition (Irvin Teh, David Shelley, Richard J. Foster), MRI data analysis (Irvin Teh, Filip Szczepankiewicz), clinical expertise (Ana‐Maria Poenar, Noor Sharrack, Erica Dall'Armellina, Sven Plein), ethics approvals (Irvin Teh, Erica Dall'Armellina, Sven Plein), drafting of manuscript (Irvin Teh, Filip Szczepankiewicz), and manuscript review (All).

## FUNDING INFORMATION

This work was supported by the British Heart Foundation, UK (PG/19/1/34076, FS/13/71/30378, PG/17/28/32943, SI/14/1/30718, CH/16/2/32089); the Swedish Research Council (2021‐04844); and the Swedish Cancer Society (22 0592 JIA). Fenglei Zhou was supported by a NIHR UCLH Biomedical Research Center grant. Jürgen E. Schneider acknowledges funding from the Wellcome Trust 219536/Z/19/Z. Ana‐Maria Poenar acknowledges funding received from the European Society of Cardiology in the form of an ESC Training Grant (000062956/2020).

## CONFLICT OF INTEREST STATEMENT

FS and IT are inventors on patents related to this study. FS declares ownership interests in Random Walk Imaging, which holds patents related to the methodology. GJMP is a director and shareholder in Bioxydyn Limited, in Quantitative Imaging Limited, and in Queen Square Analytics, companies with interests in quantitative imaging. The remaining authors declare no conflicts of interest.

## ETHICS STATEMENT

Volunteers provided informed consent. The study was carried out under approved ethics (Yorkshire & The Humber—Leeds East Research Ethics Committee 18/YH/0168; Yorkshire & The Humber—South Yorkshire Research Ethics Committee 19/YH/0324).

## Supporting information


**Figure S1.** Mean diffusivity (MD) maps in the fiber phantom. Data are shown in the central slice where fiber populations overlap, at different mean frequencies of the power spectrum of **q**(*t*) and interblock angles. Parameter values were reported in a central region of interest with radius of 5 voxels (red outline). MD is seen to decrease with increasing diffusion encoding time.
**Figure S2.** Fractional anisotropy (FA) and microscopic FA (μFA) maps in the fiber phantom. Data are shown in the central slice where fiber populations overlap, at different mean frequencies of the power spectrum of **q**(*t*) and interblock angles. FA maps are given in Rows 1 to 6 and μFA in the bottom row. FA is seen to increase with increasing diffusion encoding time and decrease with increasing interblock angle. μFA remains relatively insensitive to interblock angle.

## Data Availability

The waveform optimization constraints, code to perform optimization and subselection, along with the final waveforms used herein, are available at https://github.com/filip‐szczepankiewicz/Teh_MRM_2023. The data that support the findings of this study are available from the corresponding author upon reasonable request.
