# Supplementary material for: Cardiac q‐space trajectory imaging by motion‐compensated tensor‐valued diffusion encoding in human heart in vivo
Source: Magn Reson Med. 2023 Mar 20;90(1):150–65. doi: 10.1002/mrm.29637 (PMC10952623; doi:10.1002/mrm.29637)
Supplement: Supplementary file 1 — Figure S1. Mean diffusivity (MD) maps in the fiber phantom. Data are shown in the central slice where fiber populations overlap, at different mean frequencies of the power spectrum of q(t) and interblock angles. Parameter values were reported in a central region of interest with radius of 5 voxels (red outline). MD is seen to decrease with increasing diffusion encoding time. Figure S2. Fractional anisotropy (FA) and microscopic FA (μFA) maps in the fiber phantom. Data are shown in the central slice where fiber populations overlap, at different mean frequencies of the power spectrum of q(t) and interblock angles. FA maps are given in Rows 1 to 6 and μFA in the bottom row. FA is seen to increase with increasing diffusion encoding time and decrease with increasing interblock angle. μFA remains relatively insensitive to interblock angle. [file MRM-90-150-s001.docx]

**SUPPLEMENTARY INFORMATION**


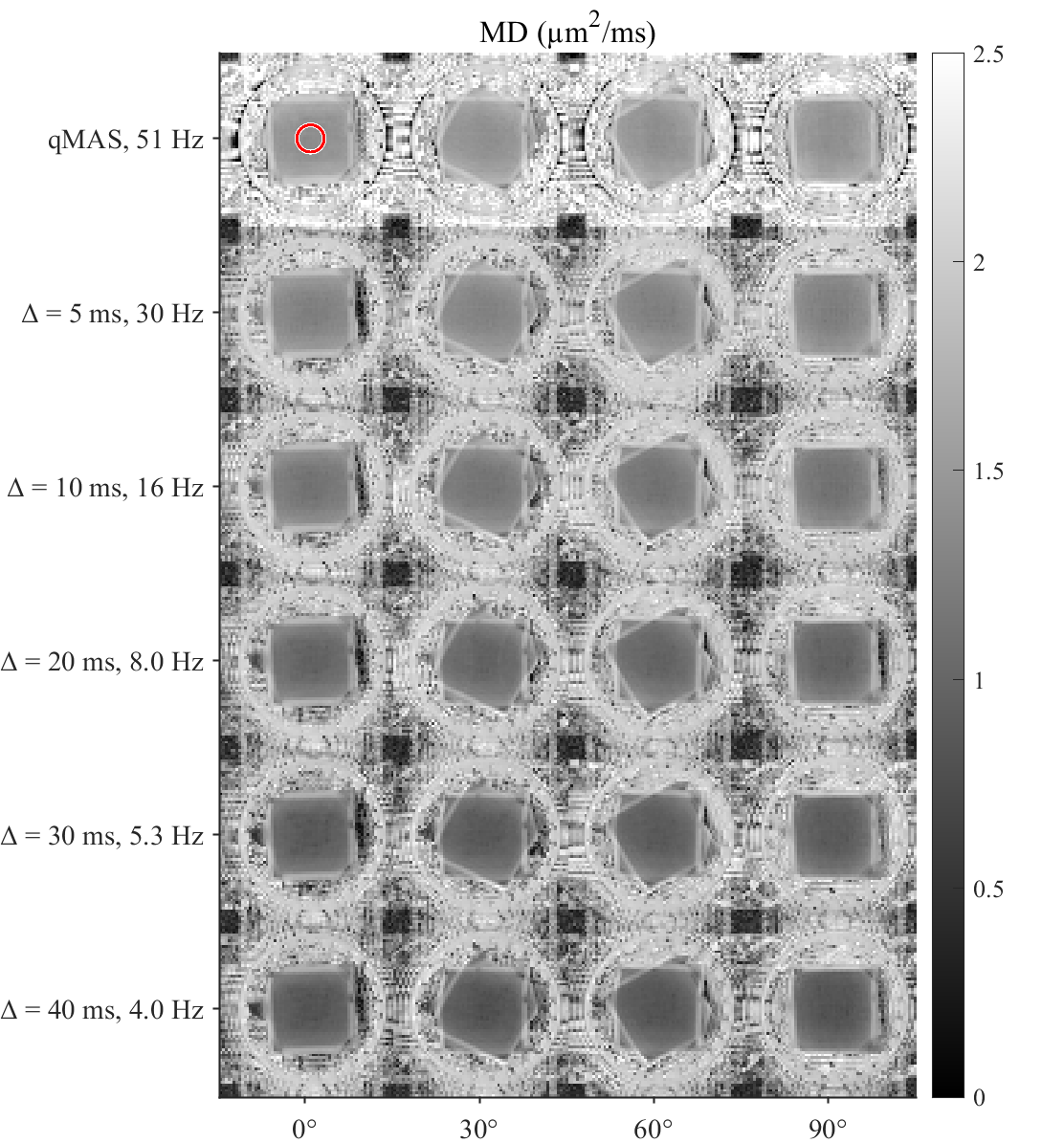


Supplementary Figure 1. Mean diffusivity (MD) maps in the fibre phantom. Data are shown in the central slice where fibre populations overlap, at different mean frequencies of the power spectrum of **q**(t) and inter-block angles. Parameter values were reported in a central region-of-interest with radius of 5 voxels (red outline). MD is seen to decrease with increasing diffusion encoding time.


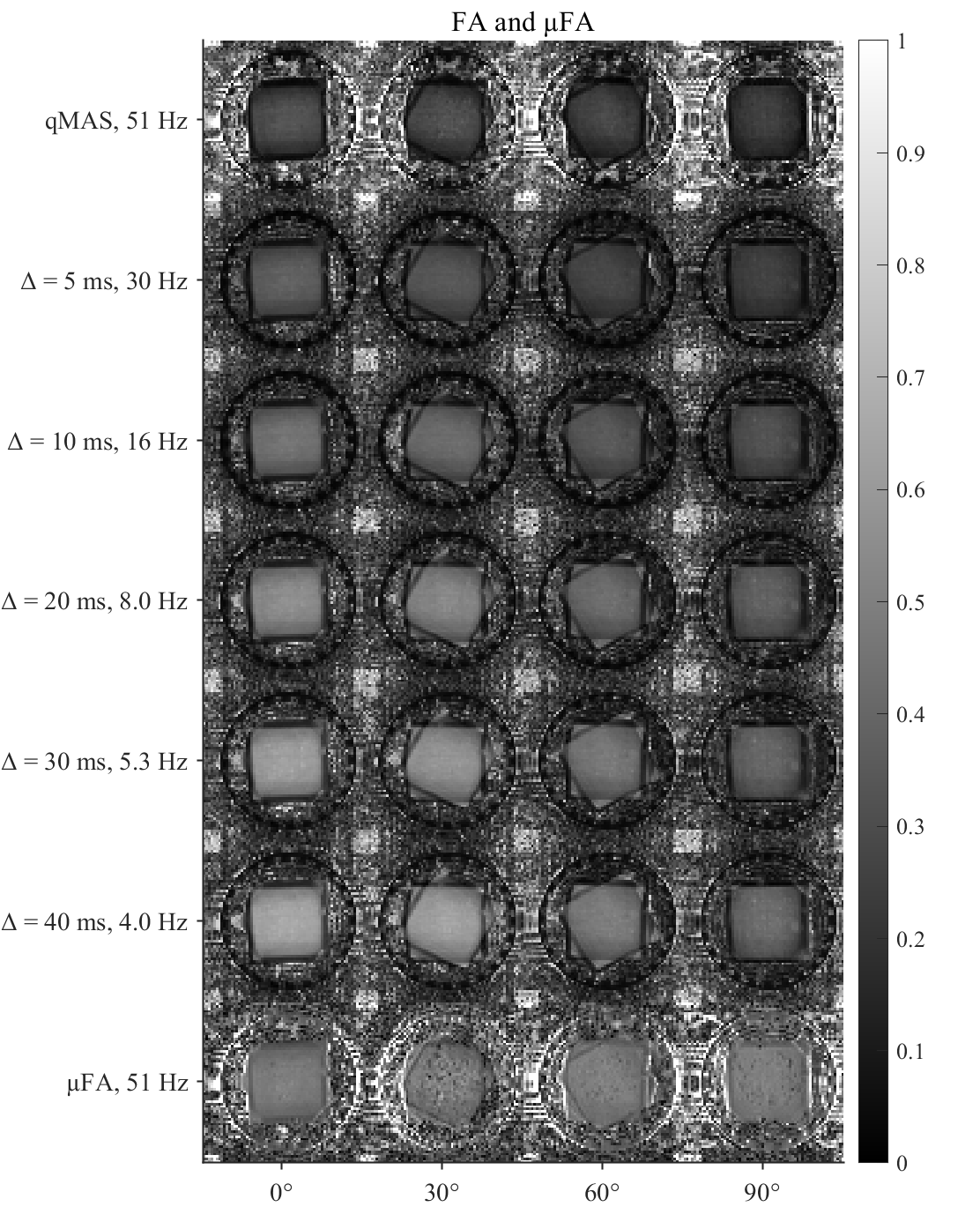


Supplementary Figure 2. Fractional anisotropy and µFA maps in the fibre phantom. Data are shown in the central slice where fibre populations overlap, at different mean frequencies of the power spectrum of **q**(t) and inter-block angles. FA maps given in Rows 1 to 6 and µFA in bottom row. FA is seen to increase with increasing diffusion encoding time, and decrease with increasing inter-block angle. µFA remains relatively insensitive to inter-block angle.
